# Editorial: Advances in veterinary 3D modeling: applications of CT, MRI, and scanning technologies

**DOI:** 10.3389/fvets.2026.1929435

**Published:** 2026-07-23

**Authors:** Ozan Gündemir, Nedžad Hadžiomerović

**Affiliations:** 1 Department of Anatomy, Faculty of Veterinary Medicine, Istanbul University-Cerrahpasa, Istanbul, Türkiye; 2 Department of Basic Sciences of Veterinary Medicine, Veterinary Faculty, University of Sarajevo, Sarajevo, Bosnia and Herzegovina

**Keywords:** 3D technology in veterinary research, diagnostic imaging, digital anatomy, imaging technologies, shape analysis, veterinary diagnostics and modeling

Advanced imaging and digital modeling tools are reshaping how anatomy is documented, taught, interpreted, and translated into clinical decision-making in veterinary science. This Research Topic shows that computed tomography (CT), magnetic resonance imaging (MRI), ultrasonography, micro-CT, three-dimensional (3D) volume rendering, three-dimensional (3D) scanning, virtual reality, and patient-specific manufacturing are not isolated technical innovations. Rather, they are becoming components of an integrated data workflow that connect diagnostic interpretation, morphometric measurement, education, surgical planning, and longitudinal monitoring. Taken together, the contributions to this Research Topic indicate a clear shift in veterinary imaging from static visualization toward quantitative, workflow-based, and patient- or species-specific applications.

A first key axis of the Research Topic is the reconstruction of species-specific anatomical reference knowledge through advanced imaging. The use of in ovo MRI to visualize and volumetrically assess the spleen in chicken embryos introduces a non-invasive framework for developmental and immunological studies in avian models (Vali et al.). A cross-sectional atlas of the tree shrew, based on micro-CT imaging and corresponding cadaveric sections, provides an anatomical reference for a research species for which detailed cross-sectional resources remain limited (Chen et al.). In dromedary camels, the combined use of CT, 3D volume-rendered CT, MRI, and ultrasonography in the pes region demonstrates how complex distal limb anatomy can be described more reliably through multimodal imaging (Hamoda et al.). Similarly, microcomputed tomographic examination of cortical and trabecular structures of the cranial cervical spine in dogs and cats provides a high-resolution reference for comparative morphology and neuro-orthopedic assessment of this anatomically challenging region (Klotz et al.).

This anatomical axis is closely linked to education. CT-based 3D models of the os cordis in wild ungulates show how digital reconstructions can help distinguish physiological variation from pathological change and support teaching in wildlife-related veterinary contexts (Matějka Košinová et al.). The integration of 3D scanning and virtual reality into meat-cut anatomy extends digital anatomy beyond structural identification to include food hygiene, professional training, and applied veterinary education (Hadžiomerović et al.). These contributions indicate that 3D modeling is moving beyond passive visual supplementation and toward interactive, measurable, and context-rich learning environments.

A second focus concerns the ability of imaging to objectify clinically subtle, complex, or anatomically difficult disease patterns. A CT-based evaluation of para-aural mineralization in dogs suggests that an uncommon mineralization pattern may serve as an imaging marker associated with chronic middle ear disease (Ma et al.). A large, retrospective, imaging-based evaluation of feline inflammatory aural polyps emphasizes the importance of video otoscopy, CT, and MRI for lesion localization, assessment of extent, and individualized surgical planning (Demirutku and Bilgiç). The diagnosis of cervical internal vertebral venous plexus thrombosis using non-contrast time-of-flight MRA expands the scope of vascular imaging diagnostics in veterinary neurology (Canejo-Teixeira et al.). CT-based measurement of the external jugular vein cross-sectional area in cats further demonstrates how imaging data can be translated directly into practical decisions about vascular access and catheter selection (Seo et al.).

This Research Topic also highlights the potential of ultrasonography and MRI to monitor dynamic biological processes. Shear-wave speed assessment of the cervix and vulvar lips in Kivircik ewes before and after parturition illustrates how elastography can quantify periparturient tissue remodeling (Günay Uçmak et al.). Longitudinal ultrasonographic measurement of placentome diameter, interpreted alongside maternal ovine placental lactogen concentrations, provides a framework for integrating structural biometry and endocrine profiling during gestation (Baykal et al.). In a porcine crush-injury model, the combination of T2-weighted Dixon MRI, two-dimsional ultrasonography, biochemical markers, and histopathology demonstrates how imaging can contribute to the temporal assessment of soft-tissue injury (Wang et al.). Ultrasonographic follow-up of the patellar ligament after cranial cruciate ligament repair in dogs further strengthens the role of ultrasound in post-operative tissue monitoring and complication assessment (Candela Andrade et al.).

A third axis is formed by morphometry, risk assessment, and patient-specific surgical planning. A CT-based morphometric evaluation of the atlanto-occipital and lateral atlanto-axial joints in small-breed dogs provides a more measurable basis for understanding the relationship between craniovertebral junction morphology and atlanto-axial instability (Pack et al.). Three-dimensional CT volumetric analysis of spinal canal volume in Bichon Frisé, Dachshund, and French Bulldog dogs links morphometric capacity to neurological severity in thoracolumbar disc herniation, highlighting the prognostic potential of quantitative imaging (Voiculeţ et al.). In canine orthopedics, 3D printing supports preoperative planning, the creation of patient-specific guides, and decision-making in complex deformity cases (Thomas et al.). The adaptation of CT imaging, iterative closest point alignment, and thin-plate spline computational modeling to customized osteosynthesis plates for multi-species fractures demonstrates how patient-specific implant design can be made more algorithmic and reproducible (Fares et al.). As a clinical extension of this trajectory, the dorsal stabilization of an occipito-atlanto-axial malformation in a Miniature Dachshund using 3D planning and a patient-specific titanium implant demonstrates the feasibility of combining advanced imaging with personalized manufacturing in complex neuro-orthopedic cases (Pajor and Czeibert).

A further strength of this Research Topic is that 3D modeling is beginning to represent not only anatomical volumes, but also movement, posture, and functional relationships over time. A CT-derived dynamic 3D horse model illustrates how whole-body pathomechanics can be visualized in relation to fetlock joint degeneration and how responses to rehabilitation or targeted training can be tracked conceptually (Miller et al.). When considered alongside the temporal-monitoring paradigms presented by Wang et al., Baykal et al., and Günay Uçmak et al., this study indicates that veterinary 3D modeling is progressing from static reconstruction toward process-oriented, functional, and clinically interpretable modeling.

Overall, this Research Topic demonstrates that veterinary 3D modeling can no longer be reduced to a single technology or disciplinary niche. CT, MRI, ultrasonography, micro-CT, 3D scanning, virtual reality, segmentation, and patient-specific manufacturing now form a shared digital language across anatomy, education, diagnosis, reproductive monitoring, orthopedics, neurology, wildlife research, and surgical planning. The future impact of this field will depend not only on the production of higher-resolution images but also on standardized segmentation, model validation, multicenter data sharing, correlation with clinical outcomes, and accessible educational resources. Collectively, these contributions position veterinary digital anatomy not merely as a technical adjunct but as an interdisciplinary infrastructure for clinical reasoning, teaching, and personalized care.

Nevertheless, it should be recognized that three-dimensional models may not always represent real anatomical structures with complete accuracy. Model accuracy is directly influenced by factors such as the resolution of the source images, slice thickness, imaging artifacts, scanning protocols, and the quality of the segmentation process. Boundary delineation errors arising during manual or automated segmentation, inter-operator variability, and compatibility issues between software platforms or file formats may limit the anatomical realism, reproducibility, and clinical applicability of these models. Therefore, future studies should focus on developing standardized imaging and segmentation protocols, validating models against anatomical specimens or clinical findings, and improving data interoperability across different software systems. In addition, extensive datasets generated through imaging and three-dimensional modeling studies are increasingly being incorporated into machine learning and artificial-intelligence-based analyses. In the future, these data may facilitate the development of reliable models capable of automatically identifying anatomical variations, detecting pathological changes, and supporting veterinarians in clinical interpretation, diagnosis, and decision-making.

